# The dipeptidyl peptidase‐4 (DPP‐4) inhibitor teneligliptin enhances brown adipose tissue function, thereby preventing obesity in mice

**DOI:** 10.1002/2211-5463.12498

**Published:** 2018-09-19

**Authors:** Kenichiro Takeda, Honami Sawazaki, Haruya Takahashi, Yu‐Sheng Yeh, Huei‐Fen Jheng, Wataru Nomura, Takeshi Ara, Nobuyuki Takahashi, Shigeto Seno, Naoki Osato, Hideo Matsuda, Teruo Kawada, Tsuyoshi Goto

**Affiliations:** ^1^ Laboratory of Molecular Function of Food Division of Food Science and Biotechnology Graduate School of Agriculture Kyoto University Uji Japan; ^2^ Research Unit for Physiological Chemistry The Center for the Promotion of Interdisciplinary Education and Research Kyoto University Kyoto Japan; ^3^ Department of Bioinformatic Engineering Graduate School of Information Science and Technology Osaka University Suita Japan

**Keywords:** beige adipocytes, brown adipocytes, dipeptidyl peptidase‐4, obesity, teneligliptin, UCP1

## Abstract

To clarify the effects of a dipeptidyl peptidase‐4 (DPP‐4) inhibitor on whole‐body energy metabolism, we treated mice fed a high‐fat diet (HFD) with teneligliptin, a clinically available DPP‐4 inhibitor. Teneligliptin significantly prevented HFD‐induced obesity and obesity‐associated metabolic disorders. It also increased oxygen consumption rate and upregulated uncoupling protein 1 (UCP1) expression in both brown adipose tissue (BAT) and inguinal white adipose tissue (iWAT), suggesting that it enhances BAT function. Soluble DPP‐4 inhibited β‐adrenoreceptor‐stimulated UCP1 expression in primary adipocytes, and this inhibition was prevented in the presence of teneligliptin, or an extracellular signal‐related kinase inhibitor. These results indicate that soluble DPP‐4 inhibits β‐adrenoreceptor‐stimulated UCP1 induction and that chronic DPP‐4 inhibitor treatment may prevent obesity through the activation of BAT function.

**Abbreviations**
BAT: brown adipose tissue
CIDEa: cell death‐inducing DFFA‐like effector A
CPT1b: carnitine palmitoyl transferase 1 B
DIO2: iodothyronine deiodinase 2
DPP‐4: dipeptidyl peptidase‐4
ELOVL3: elongation of very long chain fatty acid‐like 3
ERK: extracellular signal‐related kinase
GLP‐1: glucagon‐like peptide‐1
HFD: high‐fat diet
MCP1: monocyte chemoattractant protein 1
PAR2: protease‐activated receptor 2
PGC1α: peroxisome proliferator‐activated receptor γ coactivator 1 α
PPARα: peroxisome proliferator‐activated receptor α
Prdm16: PR domain containing 16
RER: respiratory exchange ratio
SVF: stromal vascular fraction
TNF‐α: tumour necrosis factor α
UCP1: uncoupling protein 1
WAT: white adipose tissue

Obesity is defined as a state of excessive adiposity, and it occurs when an individual's caloric intake exceeds their energy expenditure. Obesity causes excess fat accumulation not only in adipose tissues but also in other insulin‐responsive organs such as the skeletal muscle and the liver, predisposing one to the development of insulin resistance, which can lead to a range of obesity‐related metabolic disorders 1. Thus far, the molecular mechanisms underlying obesity and obesity‐related metabolic disorders have not been fully clarified, and effective therapeutic approaches are currently of general interest 2.

There are two types of adipose tissue in mammals: white adipose tissue (WAT) and brown adipose tissue (BAT). The main parenchymal cells found in these adipose depots are called adipocytes. White adipocytes store energy required for the metabolic needs of the organism, whereas brown adipocytes burn energy for thermogenesis 3. The gene responsible for nonshivering thermogenesis in brown adipocytes is uncoupling protein 1 (UCP1), a proton channel located in the mitochondrial inner membrane 3. In addition to these two types of adipocytes, recent studies have shown that there is a third kind of adipocyte, referred to as a beige adipocyte, which is a second type of thermogenic adipocyte that expresses functional UCP1 and is recruited in WAT following exposure to specific hormonal and environmental stimuli, such as cold exposure and noradrenaline, through a process called browning 3. In a transgenic mouse model, browning of WAT leads to protection against obesity and associated metabolic derangements 3, 4. Moreover, BAT activity has been shown to be inversely correlated with obesity and obesity‐related metabolic disorders, such as diabetes and hyperlipidaemia, in both rodents and humans 5, 6. Therefore, BAT activation may be a suitable target for the management of obesity and obesity‐related metabolic disorders.

Dipeptidyl peptidase‐4 (DPP‐4) is a ubiquitous transmembrane glycoprotein that cleaves N‐terminal dipeptides from a variety of substrates, including growth factors and hormones, neuropeptides and chemokines 7. Two important substrates of DPP‐4 are glucagon‐like peptide‐1 (GLP‐1) and gastric inhibitory polypeptide (GIP), which are released from the intestinal mucosa to help further stimulate postprandial insulin secretion through the so‐called ‘incretin effect’ 8. Enhanced glucose‐stimulated insulin secretion and improved glucose tolerance have been found in DPP‐4‐deficient mice 9. Because the insulinotropic effect of GLP‐1 remains active under hyperglycaemic conditions in type 2 diabetes, various DPP‐4‐inhibitors that prolong GLP‐1 activity are now in clinical use as antidiabetic drugs that act to increase postprandial insulin action 7, 8. Interestingly, mice lacking DPP‐4 are also protected against HFD‐induced obesity due to an enhancement of energy expenditure, at least partially 10. However, the molecular mechanisms underlying this anti‐obesity phenotype in DPP‐4‐deficient mice have not been fully clarified.

In this study, we investigated whether chronic treatment with teneligliptin, a clinically available DPP‐4 inhibitor, affects energy metabolism in mice fed a HFD. Teneligliptin treatment was found to prevent obesity and obesity‐associated metabolic disorders. The BAT activity in mice treated with teneligliptin was found to be higher than that in control mice. In primary adipocytes, soluble DPP‐4 inhibited β‐adrenoreceptor‐stimulated UCP1 induction, and teneligliptin could prevent this inhibition. These results indicate that long‐term treatment with DPP‐4 inhibitors could have a significant impact on body weight control and energy homeostasis by modulating BAT activity, providing a validation of DPP‐4 inhibition as a viable therapeutic option for the treatment of metabolic disorders related to diabetes and obesity.

## Materials and methods

### Materials

Teneligliptin hydrobromide hydrate (>95% purity confirmed by HPLC) was kindly provided by Mitsubishi Tanabe Pharma Corporation (Osaka, Japan). Recombinant DPP‐4, PD98059 and GB83 were purchased from Abnova (Taipei, Taiwan), Calbiochem (La Jolla, CA, USA) and Axon (Groningen, the Netherlands), respectively. Unless otherwise indicated, all other chemicals used were purchased from Sigma (St. Louis, MO, USA), Nacalai Tesque (Kyoto, Japan) or Wako (Osaka, Japan).

### Animals

For the diet‐induced obesity model, 5‐week‐old male C57BL/6N mice (Japan SLC, Shizuoka, Japan) were purchased from SLC (Hamamatsu, Japan). After 1 week of acclimatization, mice were divided into three groups: a normal diet (ND)‐treated group (10 kcal% fat; Oriental Yeast, Tokyo, Japan), a HFD‐treated group (60 kcal% fat; Research Diets, MO, USA) and a HFD supplemented with teneligliptin‐ (80 mg·kg^−1^·day^−1^; mixed in the drinking water) or CL316243 (a β3‐adrenergic agonist)‐treated group (0.5 mg·kg^−1^·day^−1^; intraperitoneal injection). Animals were housed under a constant 12‐h light/dark cycle with *ad libitum* access to food and water. Obese diabetic db/db mice and lean control mice were purchased from SLC. All animal care and experimental procedures were approved by the Kyoto University Animal Care Committee.

### Cell culture

C3H10T1/2 cells (10T1/2) (Dainippon Sumitomo Pharma, Osaka, Japan) and primary cultured pre‐adipocytes, isolated from male C57BL/6N mice as described previously 11, were cultured in DMEM supplemented with 10% FBS, 100 U·mL^−1^ penicillin and 100 μg·mL^−1^ streptomycin at 37 °C under a humidified 5% CO_2_ atmosphere. The 10T1/2 cells and primary cultured pre‐adipocytes were induced to differentiate into adipocytes as described previously 12, 13. Six to eight days after the induction of differentiation, the cells were treated with DPP‐4 and several inhibitors with or without isoproterenol or forskolin.

### Plasma characteristics and hepatic lipid analysis

Plasma glucose and triglycerol levels, as well as hepatic lipid content, were enzymatically determined as described previously 14.

### RNA preparation and quantification of gene expression

Total RNA was prepared from animal tissues and cultured cells using Sepasol‐RNA I Super G (Nacalai Tesque) in accordance with the manufacturer's protocol. Total RNA was reverse transcribed using M‐MLV reverse transcriptase (Promega, Madison, WI, USA). To quantify mRNA expression, real‐time RT‐PCR was performed using a Light Cycler System (Roche Diagnostics, Mannheim, Germany) using SYBR Green fluorescence signals. The mRNA expression levels were normalized to 36B4 mRNA levels for quantification. The oligonucleotide primers used in this study are listed in Table 1.

**Table 1:** Oligonucleotide primers used for mRNA analysis

Gene	Forward primer	Reverse primer	Gene ID
*36B4*	TCCTTCTTCCAGGCTTTGGG	GACACCCTCCAGAAAGCGAG	11837
*Adipoq*	TACAACCAACAGAATCATTATGACGG	GAAAGCCAGTAAATGTAGAGTCGTTGA	11450
*F4/80*	TTTCCTCGCCTGCTTCTTC	CCCCGTCTCTGTATTCAACC	13733
*Mcp1*	GACCCCAAGAAGGAATGGGT	ACCTTAGGGCAGATGCAGGT	20296
*Tnf‐a*	ACATCAGATCATCTTCTCAAAATTC	GTGTGGGTGAGGAGCACGTAGT	21926
*Cd11c*	TGGGGTTTGTTTCTTGTCTTG	GCCTGTGTGATCGCCACATTT	16411
*Ucp1*	CAAAGTCGCCCTTCAGATCC	AGCCGGCTGAGATCTTGTTT	22227
*Cpt1b*	CTGTTAGGCCTCAACACCGAAC	CTGTCATGGCTAGGCGGTACAT	12895
*Dio2*	AGCCCACATGTAACCAGCACCGGA	CAGTCGCAC TGGCTCAGGAC	13371
*Pgc1a*	CCCTGCCATTGTTAAGACC	TGCTGCTGTTCCTGTTTTC	19017
*Cidea*	ATCACAACTGGCCTGGTTACG	TACTACCCGGTGTCCATTTCT	12683
*Elovl3*	AAGGACATGAGGCCCTTTTT	AAGATTGCAAGGCAGAAGGA	12686
*Prdm16*	CAGCACGGTGAAGCCATTC	GCGTGCATCCGCTTGTG	70673
*Ppara*	TCGCGTACGGCAATGGCTTT	CTCTTCATCCCCAAGCGTAGGAGG	19013
*Dpp4*	CAGCTCATCCTCTAGTGCGG	AGGTGAAGTGAGGTTCTGCG	13482

### Western blotting

Western blotting was performed as described previously 12, 13 using antibodies against extracellular signal‐related kinase (ERK), phosphorylated ERK, cytochrome oxidase complex 4 (COX4) (Cell Signalling Technology, Danvers, MA, USA) and UCP1 (Sigma).

### Measurement of oxygen consumption, RER and locomotor activity

Oxygen consumption and respiratory exchange ratio (RER) were measured using an indirect calorimetry system (Columbus, OH, USA) as described previously 15. Locomotor activity was measured using an Actimo‐S (Shinfactory, Fukuoka, Japan).

### Separation of the stromal vascular fraction and the adipocyte fraction

The stromal vascular fraction (SVF) and the adipocyte fraction were separated by collagenase digestion of epididymal WAT (eWAT) isolated from male C57BL/6 mice, as described previously 16.

### Histological analysis

Adipose tissues and the liver were fixed in 4% paraformaldehyde and then embedded in paraffin. The tissues were cut into 5–6 μm sections using a microtome and placed on microscope slides (Matsunami Glass, Osaka, Japan). The paraffin‐embedded sections were then stained with haematoxylin and eosin. Adipocyte sizes were measured using image analysis program within the ebimage package of R/Bioconductor 14.

### Luciferase assay

Uncoupling protein 1 promoter activity was determined using a luciferase reporter assay. The luciferase reporter vector, encoding luciferase under the control of the 3.8‐kb portion of the 5′‐flanking region of the mouse UCP1 gene (pUCP1‐pro‐Luc), and a Dual‐Luciferase Reporter Gene Assay system (Promega) were used as described previously 12, 13.

### Statistical analysis

The data are presented as the mean + standard error (SE). Statistical analysis was performed with a Student's *t*‐test or one‐way ANOVA followed by a Tukey–Kramer test to evaluate the statistical significance of differences.

## Results

### Teneligliptin treatment prevents HFD‐induced obesity

**Figure 1:**
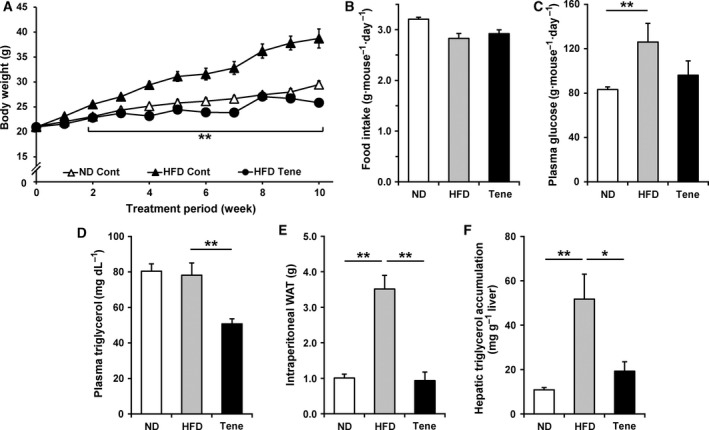
Effects of teneligliptin treatment on HFD‐induced obesity and obesity‐induced metabolic disorders. Teneligliptin was administered in the drinking water (80 mg·kg^−1^·day^−1^), for a total of 10 weeks, to male 6‐week‐old C57BL/6N mice also fed a HFD. A and B: Body weight gain (A) and food intake (B) were measured during the administration period. C–F: After 10 weeks of treatment, plasma glucose (C) and triglycerol (D) levels, intraperitoneal WAT weight (E) and hepatic triglycerol accumulation levels (F) were determined. Plasma glucose and triglycerol and hepatic triglycerol levels were determined enzymatically. HFD, high‐fat diet; ND, normal diet; Tene, teneligliptin plus HFD. All the values are means + SE (*n* = 5–8). * *P* < 0.05, ** *P* < 0.01.

To investigate whether teneligliptin treatment affects energy metabolism, we administered teneligliptin mixed in drinking water (80 mg·kg^−1^·day^−1^) to mice fed an HFD. As shown in Fig. 1A, HFD feeding significantly induced body weight gain in mice compared with normal diet (ND) feeding. However, teneligliptin treatment completely suppressed this HFD‐induced body weight gain (Fig. 1A and Table 2), in spite of there being no significant changes in food intake (Fig. 1B and Table 2). After ten weeks of teneligliptin treatment, plasma glucose levels tended to be reduced (*P* = 0.09) (Fig. 1C) and plasma triglycerol levels (Fig. 1D) were significantly reduced in HFD mice treated with teneligliptin compared with the HFD control group. The weight of intraperitoneal white adipose tissue (WAT) was markedly increased by HFD feeding, whereas teneligliptin treatment almost completely blocked this HFD‐induced intraperitoneal WAT expansion (Fig. 1E and Table 2). Furthermore, teneligliptin significantly inhibited HFD‐induced hepatic lipid accumulation (Fig. 1F). These data indicate that teneligliptin treatment can inhibit diet‐induced body weight gain and lipid accumulation not only in adipose tissue, but also in nonadipose tissues, such as the liver.

**Table 2:** Body and tissue weights of mice treated with or without teneligliptin for 10 weeks

Diet	ND	HFD	
Treatment	Vehicle	Vehicle	Teneligliptin
Food intake (g·day^−1^)	3.20 ± 0.04	2.83 ± 0.10	2.92 ± 0.15
Body weight (g)	29.5 ± 0.74	38.7 ± 1.91*	25.9 ± 0.69#
Tissue weight (g/100 g body weight)			
Inguinal WAT	1.045 ± 0.074	2.418 ± 0.242*	1.320 ± 0.189#
Epididymal WAT	1.692 ± 0.192	4.285 ± 0.363*	1.882 ± 0.504#
Mesenteric WAT	0.904 ± 0.072	2.146 ± 0.289*	0.674 ± 0.137#
Perirenal WAT	0.799 ± 0.094	2.408 ± 0.203*	0.998 ± 0.231#
Interscapular BAT	0.311 ± 0.015	0.375 ± 0.037	0.261 ± 0.015#
Gastrocnemius	1.020 ± 0.016	0.844 ± 0.042*	1.057 ± 0.033#
Liver	4.749 ± 0.072	4.099 ± 0.319	3.751 ± 0.262
Kidney	1.164 ± 0.014	1.018 ± 0.047*	1.278 ± 0.055#

The values are shown as means ± SE (*n* = 6–10).

*P < 0.05 compared with ND vehicle.

# *P* < 0.05 compared with HFD vehicle.

### Teneligliptin treatment prevents HFD‐induced WAT dysfunction

**Figure 2:**
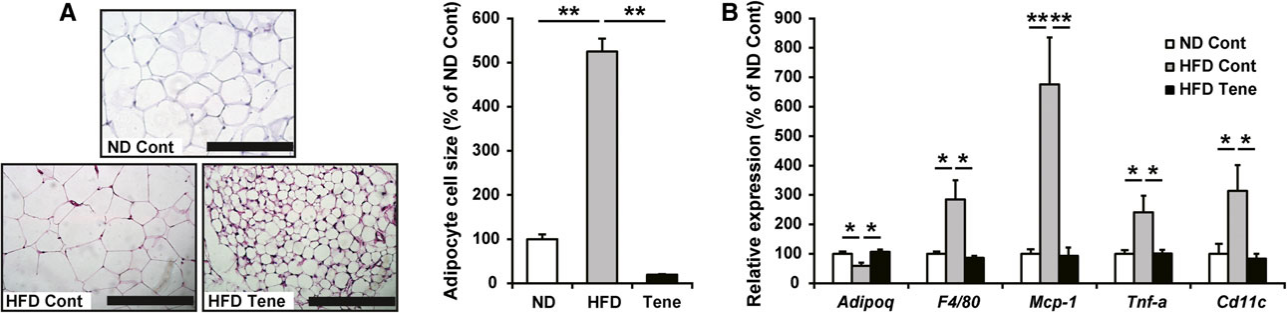
Effects of teneligliptin treatment on HFD‐induced WAT dysfunction. Teneligliptin was administered in the drinking water (80 mg·kg^−1^·day^−1^), for a total of 10 weeks, to male 6‐week‐old C57BL/6N mice also fed a HFD. (A) iWAT samples embedded in the paraffin were cut into 6‐μm sections, and each section was stained with haematoxylin and eosin. Representative images of iWAT sections are shown (the scale bars in the panels represent 200 μm). The average adipocyte size was determined by image analysis. All values are means + SE (*n* = 7–10). (B) mRNA expression levels of genes related to chronic inflammation in eWAT were determined by real‐time RT‐PCR. HFD, high‐fat diet; ND, normal diet; Tene, teneligliptin plus HFD. All the values are means + SE (*n* = 8–10). *P < 0.05, **P < 0.01.

Next, we investigated whether teneligliptin treatment had preventive effects on obesity‐induced WAT dysfunction. In a histochemical analysis of inguinal WAT (iWAT), we found that adipocyte hypertrophy was induced by HFD feeding (Fig. 2A). However, teneligliptin treatment completely suppressed this HFD‐induced adipocyte hypertrophy. Interestingly, in spite of HFD‐feeding, the adipocyte cell size in the iWAT of mice treated with teneligliptin was smaller than that in the iWAT of mice fed ND (Fig. 2A). In obese adipose tissue, infiltration of macrophages induces chronic inflammation, which leads to adipocyte dysfunction, such as that observed in insulin resistance 1. Therefore, we measured the mRNA expression levels of genes related to chronic inflammation in obese adipose tissue. As shown in Fig. 2B, HFD feeding significantly reduced the mRNA expression levels of *Adipoq*, an anti‐inflammatory adipokine, whereas it enhanced the mRNA expression level of proinflammatory cytokines (monocyte chemoattractant protein‐1 (*Mcp‐1*) and tumour necrosis factor α (*Tnf‐*α)) and the macrophage marker genes (*F4/80* and *Cd11c*), suggesting that HFD‐induced chronic inflammation in eWAT in this animal model. Importantly, teneligliptin treatment almost completely prevented these HFD‐induced changes in gene expression, suggesting that teneligliptin treatment could suppress the onset of obesity‐induced chronic inflammation in eWAT.

### Teneligliptin treatment enhanced UCP1 expression in BAT and iWAT, leading to an enhancement of energy expenditure

**Figure 3:**
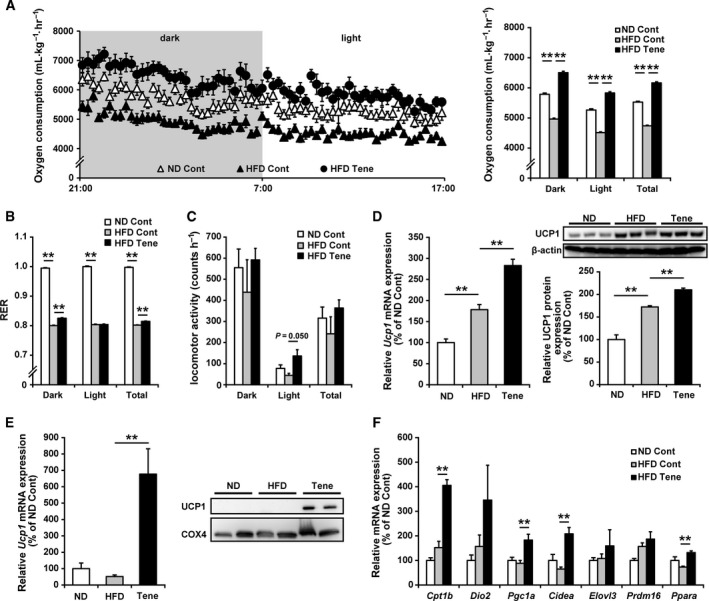
Effects of teneligliptin treatment on whole‐body energy expenditure and BAT function in BAT and iWAT. Teneligliptin was administered in the drinking water (80 mg·kg^−1^·day^−1^), for a total of 10 weeks, to male 6‐week‐old C57BL/6N mice also fed a HFD. A–C: Ten weeks after initial treatment, oxygen consumption rate (A) and RER (B) were measured by indirect calorimetry, and locomotor activity (C) was measured using an infrared sensor under the fed condition for 20 h (dark phase: 10 h; light phase: 10 h). (D) The mRNA and protein expression levels of UCP1 in BAT were determined by real‐time RT‐PCR and immunoblotting, respectively. (E,F) The mRNA and protein expression levels of UCP1 (E) and the mRNA expression levels of genes related to BAT function (F) in iWAT were determined by immunoblotting (protein expression levels) and real‐time RT‐PCR (mRNA expression levels). HFD, high‐fat diet; ND normal diet; Tene, teneligliptin plus HFD. All the values are means + SE (*n* = 7–10). *P < 0.05, **P < 0.01.

To understand why teneligliptin treatment had a preventive effect on HFD‐induced obesity, we measured the oxygen consumption rate of animals using an indirect calorimeter. As shown in Fig. 3A, teneligliptin treatment completely prevented the HFD‐induced reduction in oxygen consumption rate seen in both the dark and light phases. In fact, teneligliptin treatment slightly increased the RER in the dark phase (Fig. 3B). HFD‐feeding tended to decrease locomotor activity and teneligliptin treatment tended to prevent this reduction in locomotor activity, but these changes were not significant (Fig. 3C), suggesting that teneligliptin treatment enhanced energy expenditure without inducing hyperactivity. Next, we investigated whether there were changes in BAT function that were related to the teneligliptin treatment‐induced enhancement of energy expenditure. Teneligliptin treatment significantly increased the mRNA and protein expression levels of uncoupling protein 1 (UCP1) in BAT (Fig. 3D). Moreover, teneligliptin treatment also markedly increased UCP1 mRNA and protein expression levels in iWAT (Fig. 3E). Based on the Cp values from quantitative PCR, under the normal diet condition, the *Ucp1* mRNA levels are about 5000‐fold higher in BAT than in iWAT (average Cp values in *Ucp1* quantification are 12.27 [normal diet in BAT] and 22.94 [normal diet in iWAT]). We also confirmed that the mRNA expression levels of several genes related to BAT function (namely, carnitine palmitoyl transferase 1 B (*Cpt1b*), peroxisome proliferator‐activated receptor γ coactivator 1 α (*Pgc1a*), cell death‐inducing DFFA‐like effector A (*Cidea*) and peroxisome proliferator‐activated receptor α (*Ppara*)) also significantly increased in the iWAT of mice treated with teneligliptin (Fig. 3F), suggesting that teneligliptin treatment increased BAT function in both BAT and iWAT.

### A soluble form of DPP‐4 inhibited β‐adrenoreceptor agonist‐induced *Ucp1* expression, and teneligliptin could inhibit this in cultured cells

**Figure 4:**
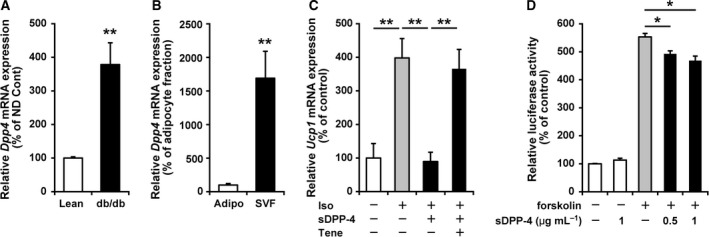
Effects of the soluble form of DPP‐4 on *Ucp1* expression in cultured white adipocytes. A and B: Expression of *Dpp4* mRNA in eWAT. The mRNA expression levels of *DPP4* in eWAT from lean control and obese diabetic db/db mice (A), and in the adipocyte fraction (Adipo) and SVF fraction from eWAT (B), were determined by real‐time RT‐PCR. C: 10T1/2 cells were induced to differentiate into adipocytes. Six days after the induction of differentiation, cells were treated with or without 1 μm isoproterenol (Iso), 1 μg·mL ^−1^ sDPP‐4 and 100 μm teneligliptin (Tene) for 8 h. After cDNA preparation, *Ucp1* mRNA expression levels were determined by real‐time RT‐PCR. D: Effects of sDPP‐4 on 1 μm forskolin‐induced activation of the *Ucp1* promoter. 10T1/2 cells were transfected with pUCP1‐pro‐Luc and incubated in medium, with or without sDPP‐4, followed by treatment with 0.5 or 1 μm forskolin, with or without sDPP‐4, for an additional 8 h. All the data are shown as mean + SE (*n* = 5–6). *P < 0.05, **P < 0.01.

As previously described 17, the mRNA expression levels of *Dpp4* in eWAT were higher in obese and diabetic mice than in lean control mice (Fig. 4A). In eWAT, *Dpp4* mRNA was predominantly expressed in the SVF compared to the adipocyte fraction (Fig. 4B). Recently, it has been shown that DPP‐4 is one of the adipokines potentially linking obesity to metabolic syndrome 17. Therefore, we investigated whether the soluble form of DPP‐4 (sDPP‐4), derived from the SVF, affects *Ucp1* expression levels in adipocytes in a paracrine fashion. Although the addition of sDPP‐4 did not affect basal *Ucp1* expression levels (data not shown), sDPP‐4 completely inhibited β‐adrenoreceptor agonist (isoproterenol)‐induced *Ucp1* mRNA expression (Fig. 4C) in 10T1/2 adipocytes. Interestingly, cotreatment with teneligliptin almost completely prevented this sDPP‐4‐mediated inhibition of *Ucp1* expression in 10T1/2 adipocytes (Fig. 4C). Using a luciferase reporter under the control of the *Ucp1* promoter region in 10T1/2 cells, we observed that sDPP‐4 partially but significantly inhibited forskolin‐induced *Ucp1* promoter activation (Fig. 4D). These results indicate that sDPP‐4 inhibits β‐adrenoreceptor agonist‐induced *Ucp1* expression through the suppression of *Ucp1* promoter activation at least partially and that teneligliptin can prevent this inhibition.

### DPP‐4 inhibits β‐adrenoreceptor agonist‐induced *Ucp1* expression via the activation of ERK signalling

**Figure 5:**
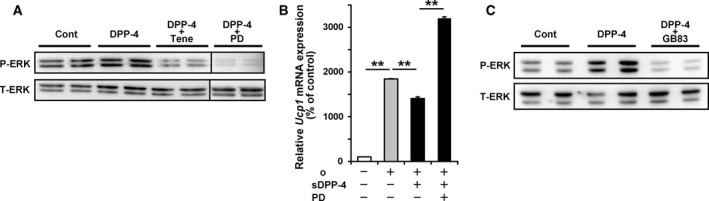
Dipeptidyl peptidase‐4 inhibits β‐adrenoreceptor agonist‐induced *Ucp1* expression through activation of ERK signalling. Mouse primary pre‐adipocytes, isolated from iWAT, were induced to differentiate into adipocytes for 6–8 days. (A) ERK protein levels (phosphorylated ERK, P‐ERK and total ERK, T‐ERK) were analysed in adipocytes treated with or without 1 μg·mL ^−1^ sDPP‐4, 100 μm teneligliptin (Tene) and 20 μm PD98509 (PD). (B) Adipocytes were incubated in the presence or absence of sDPP‐4 and PD98509 (PD) for 12 h. The cells were then treated with or without sDPP‐4, PD98509 and/or isoproterenol (Iso) for 8 h, after which the *Ucp1* mRNA levels were determined. All the values represent the mean + SE (*n* = 4–6). **P < 0.01. (C) ERK protein levels were analysed in adipocytes treated with or without sDPP‐4 and 10 μm GB83 (a PAR2 inhibitor).

It has been reported that sDPP‐4 affects target cellular functions via the activation of ERK and that a DPP‐4 inhibitor can inhibit DPP4‐mediated ERK activation 18, 19. In primary white adipocytes, the addition of sDPP‐4 enhanced the phosphorylation levels of ERK, and cotreatment with teneligliptin suppressed this sDPP‐4‐enhanced ERK phosphorylation (Fig. 5A), suggesting that sDPP‐4 activates ERK signalling in adipocytes and that teneligliptin could prevent this. Cotreatment with PD98059, a mitogen‐activated protein kinase kinase inhibitor, completely inhibited sDPP‐4‐induced ERK phosphorylation (Fig. 5A). Importantly, PD98059 treatment also abolished the inhibitory effect of sDPP‐4 on β‐adrenoreceptor agonist‐induced *Ucp1* mRNA induction (Fig. 5B), suggesting that ERK activation is important for the inhibitory effect of sDPP‐4 on β‐adrenoreceptor agonist‐induced *Ucp1* induction. PD98059 treatment increased *Ucp1* mRNA expression above the basal level. This might be caused by the PD98059‐mediated inhibition of basal ERK phosphorylation (Fig. 5A). Previous reports have shown that protease‐activated receptor 2 (PAR2) is an important mediator in this sDPP‐4‐induced ERK activation 19. Cotreatment with GB83 (a PAR2 inhibitor) markedly inhibited sDPP‐4‐induced ERK phosphorylation in primary white adipocytes (Fig. 5C), suggesting that PAR2 activation in adipocytes is important for sDPP‐4‐induced ERK activation.

### Short‐time treatment with teneligliptin increased UCP1 expression in BAT but not in iWAT

**Figure 6:**
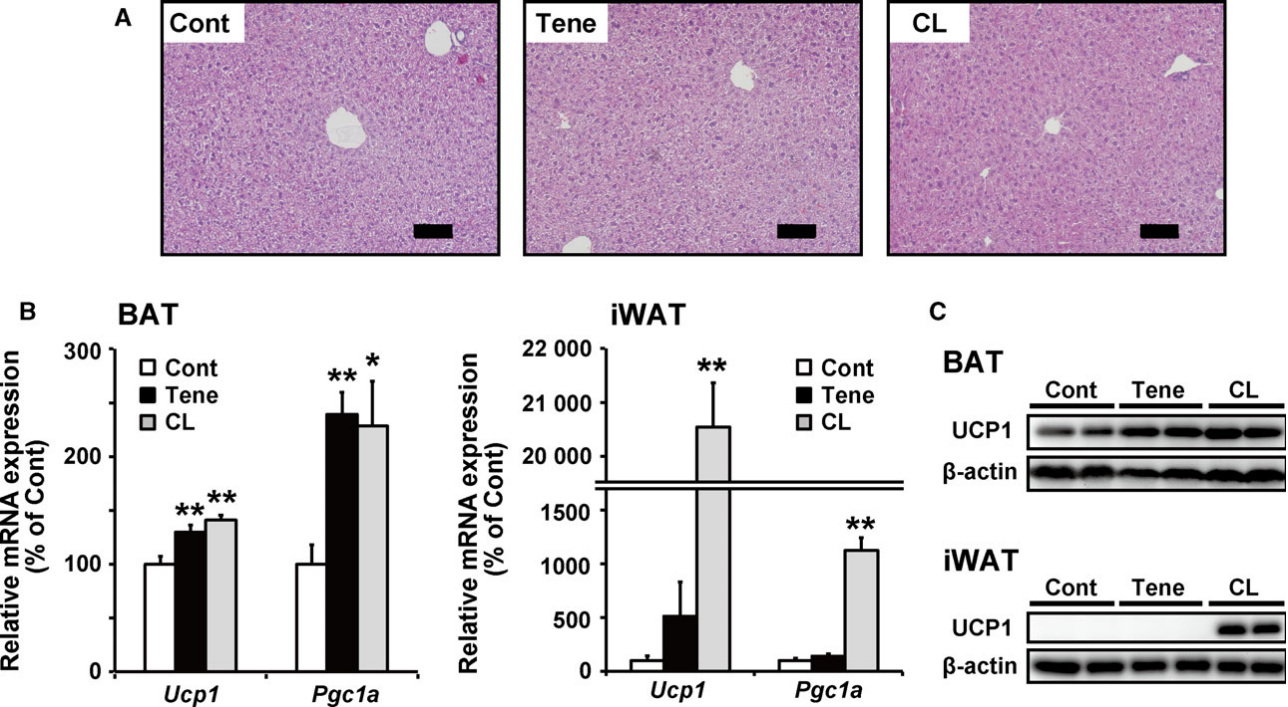
Effects of teneligliptin or CL316243 treatment on BAT function in BAT and iWAT. For 7 days, teneligliptin and CL316243 were administered in the drinking water (80 mg·kg^−1^·day^−1^) and intraperitoneal injection (0.5 mg·kg^−1^·day^−1^) to male 6‐week‐old C57BL/6N mice fed an HFD, respectively. (A) Liver samples embedded in the paraffin were cut into 5‐μm sections, and each section was stained with haematoxylin and eosin. Representative images of liver sections are shown (the scale bars in the panels represent 100 μm). (B) The mRNA expression levels of *Ucp1* and *Pgc1a* in BAT and iWAT were determined by real‐time RT‐PCR. (C) The protein levels of UCP1 in BAT and iWAT were determined by immunoblotting. All the values are means + SE (*n* = 6–9). *P < 0.05, **P < 0.01 compared with control.

Finally, to investigate whether short‐time treatment with teneligliptin affects BAT function in BAT and iWAT, we treated mice fed HFD with teneligliptin (80 mg·kg^−1^·day^−1^) or CL316243, a β3‐adrenergic agonist (0.5 mg·kg^−1^·day^−1^) for seven days. Whereas treatment with teneligliptin and CL316243 could decrease plasma glucose and triglycerol levels, they did not affect body weight and adipose tissues and liver weight (Table 3). No visible changes were observed in hepatic histochemical analysis (Fig. 6A) by teneligliptin and CL316243 treatment, and hepatic lipid accumulation levels were not changed (Table 3). As shown in Fig. 6B, *Ucp1* and *Pgc1a* mRNA expression levels in BAT were induced by both teneligliptin and CL316243 treatment, but only teneligliptin treatment failed to increase *Ucp1* and *Pgc1a* in iWAT. Similar results were obtained in the case of UCP1 protein expression levels (Fig. 6C). These results indicated that teneligliptin treatment first induces UCP1 expression in BAT, and induction of UCP1 expression levels in iWAT needs prolonged treatment.

**Table 3:** Metabolic characteristics of mice treated with or without teneligliptin or CL316243 for 7 days

Treatment	Control	Teneligliptin	CL316243
Food intake (g·day^−1^)	2.30 ± 0.11	2.11 ± 0.08	2.25 ± 0.14
Body weight (g)	22.6 ± 0.50	21.5 ± 0.37	21.6 ± 0.48
Tissue weight (g/100 g body weight)			
Inguinal WAT	1.58 ± 0.13	1.48 ± 0.11	1.38 ± 0.11
Interscapular BAT	0.36 ± 0.03	0.31 ± 0.01	0.34 ± 0.01
Liver	4.73 ± 0.08	4.49 ± 0.08	4.64 ± 0.10
Plasma glucose (mg·dL^−1^)	198 ± 6.2	177 ± 7.6*	173 ± 8.2*
Plasma triglycerol (mg·dL^−1^)	143 ± 10.1	110 ± 5.3*	102 ± 9.3*
Hepatic triglycerol (mg·g^−1^ liver)	50.4 ± 4.74	53.7 ± 5.21	46.4 ± 3.57

The values are shown as means ± SE (*n* = 6–9).

*P < 0.05 compared with control.

## Discussion

Regulation of BAT activity may represent an attractive target for therapies that are aimed at raising energy expenditure to counteract obesity and obesity‐associated metabolic disorders 20. In this study, we showed that teneligliptin enhances brown adipose tissue function in both BAT and iWAT leading to the prevention of obesity in mice. Consistent with this study, recent studies have indicated that mice chronically treated with DPP‐4 inhibitors showed decreased adiposity and body weight 21, 22, 23. Moreover, DPP‐4‐deficient mice are protected against HFD‐induced obesity via the enhancement of energy expenditure, at least partially 10. However, clinical treatment with DPP‐4 inhibitors has not been reported to have weight‐reducing effects in type 2 diabetic patients. In adult humans, the contribution of BAT activity to whole‐body energy metabolism appears to be relatively small compared to that in rodents and the BAT activity in adult humans differs substantially between individuals 24. Saito *et al*. have reported that a single oral ingestion of capsinoids increases energy expenditure in human individuals with metabolically active BAT, but not in those without 24. Diabetic status has also been reported to be negatively associated with the prevalence of BAT 25, suggesting that the beneficial effect of a BAT activator might be more limited in diabetic patients than in healthy individuals. Moreover, vildagliptin has been reported to enhance energy expenditure during intraduodenal lipid infusion in healthy men 26. Therefore, although further investigation is important, the potential utility of DPP‐4 inhibitors for the treatment of a broad spectrum of metabolic disorders related to obesity via the activation of BAT function in humans is still of interest.

Recent studies have shown that β‐adrenoreceptor‐induced activation of BAT function is suppressed under chronic inflammatory conditions induced by an increase in infiltrated macrophages in obese adipose tissue 27, 28, 29, 30. Consistent with this previous report, *Dpp4* expression levels in WAT have also been shown to be elevated in obese mice 17. In this study, *Dpp4* expression levels were higher in the SVF than in the adipocyte fraction. Therefore, increased infiltration of macrophages in which *Dpp4* is highly expressed might be pathophysiologically important for the suppression of β‐adrenoreceptor‐mediated UCP1 upregulation in obese adipose tissue. Moreover, treatment with DPP‐4 inhibitors has been shown to attenuate obesity‐induced chronic inflammation in WAT 21, 31. DPP‐4 inhibitor‐mediated suppression of chronic inflammation in obese adipose tissue might therefore be important for DPP‐4 inhibitor‐induced activation of BAT function.

Approximately 90% of DPP‐4 activity in human serum is associated with sDPP‐4, suggesting that serum sDPP‐4 reflects circulating DPP‐4 activity 32. As circulating DPP‐4 levels in human are closely associated with fat mass and type 2 diabetes mellitus 17, 33, the hormonal function of sDPP‐4 might be important. In this study, we showed that addition of sDPP‐4 inhibited β‐adrenoreceptor‐stimulated UCP1 upregulation via the activation of ERK signalling in a similar fashion to that seen for the proinflammatory cytokines, TNF‐α and interleukin‐1β 12, 13. sDPP‐4‐induced ERK activation in adipocytes appears to be mediated by PAR2, which has been shown to be important for sDPP‐4‐induced ERK activation in smooth muscle cells 19. On the other hand, it has been reported that hypothalamic activation of GLP‐1 receptor signalling stimulates BAT thermogenesis and browning, independent of nutrient intake 34, 35. Moreover, cardiac natriuretic peptides have also been shown to induce the BAT thermogenic program 36. Because both GLP‐1 and brain natriuretic peptide are cleaved and inactivated by DPP‐4 37, teneligliptin‐mediated suppression of cleavage of these hormones might contribute to BAT activation stimulated by teneligliptin.

Dipeptidyl peptidase‐4 inhibitor could increase endogenous blood levels of active incretins, leading to the stimulation of insulin secretion. Although insulin is an anabolic hormone, in this study, DPP‐4 inhibitor treatment showed rather catabolic than anabolic effect. The clear reason why these opposite observations occurred is unknown, but DPP‐4 substrate has shown to be not only incretins but also variety of growth factors and hormones, neuropeptides and chemokines 7. Moreover, GLP‐1 and GIP have been showed to have many distinct actions besides in the pancreas 38. Therefore, the whole‐body phenotype caused by the treatment of DPP‐4 inhibitor did not seem to be reflected by only insulin action. Actually, whereas DPP‐4‐deficient mice showed enhanced glucose‐stimulated insulin secretion, they also showed lean phenotype under HFD condition 9, 10.

**Figure 7:**
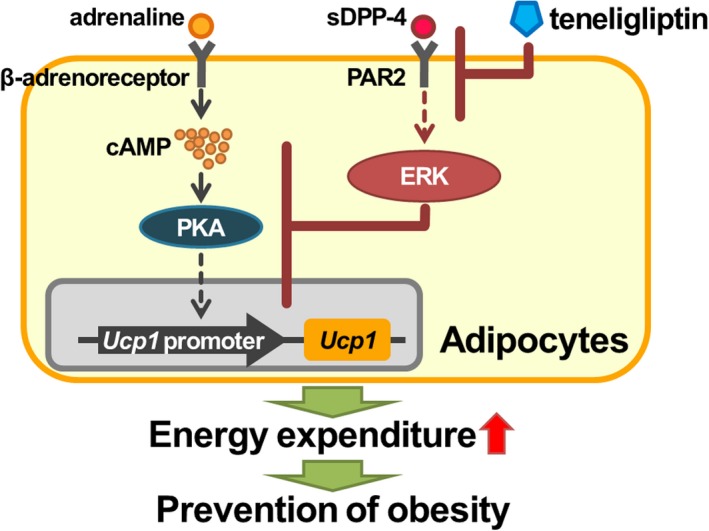
Proposed schema of preventive effect of teneligliptin on obesity in adipocytes. sDPP‐4‐mediated ERK activation, acting through PAR2, suppresses β‐adrenoreceptor‐stimulated UCP1 upregulation in adipocytes, and teneligliptin treatment could prevent this suppression, suggesting that this mechanism might be related to the teneligliptin‐induced activation of BAT function *in vivo*. Teneligliptin‐activated BAT function enhances energy expenditure, leading to the prevention of obesity.

In summary, this study demonstrated that teneligliptin treatment could prevent HFD‐induced obesity, and obesity‐associated metabolic disorders, through an increase in BAT function. sDPP‐4‐mediated ERK activation, acting through PAR2, suppresses β‐adrenoreceptor‐stimulated UCP1 upregulation in adipocytes, and teneligliptin treatment could prevent this suppression, suggesting that this mechanism might be related to the teneligliptin‐induced activation of BAT function *in vivo* (Fig. 7). These findings indicate that chronic treatment with DPP‐4 inhibitors can have a significant impact on body weight control and energy homeostasis by modulating BAT activity, providing validation of DPP‐4 inhibition as a viable therapeutic option for the treatment of metabolic disorders related to diabetes and obesity.

## Author contributions

TK and TG drafted and designed the study. KT, HS, SS and TG performed the experiments and analysed the data. KT, HS, HT, YSY, HFJ, WN, TA, NT, SS, NO, HM, TK and TG discussed and interpreted the data. HS, SS and TG wrote the draft of the manuscript HT, HFJ, NW, TA, HM, TK and TG edited the manuscript. TG supervised the study.

## Conflict of interest

The authors declare no conflict of interest.
